# Can stoic training develop medical student empathy and resilience? A mixed-methods study

**DOI:** 10.1186/s12909-022-03391-x

**Published:** 2022-05-03

**Authors:** Megan E. L. Brown, Alexander MacLellan, William Laughey, Usmaan Omer, Ghita Himmi, Tim LeBon, Gabrielle M. Finn

**Affiliations:** 1 Health Professions Education Unit, Hull York Medical School, University of York, York, UK; 2 Department of Psychology, University of Bath, Bath, UK; 3 Psychotherapist, London, UK; 4 School of Medical Sciences, Faculty of Biology, Medicine and Health, The University of Manchester, Manchester, UK

**Keywords:** Stoic training, Empathy, Resilience, Burnout, Empathic erosion, Medical education, Medical students, Psychological training, Intervention

## Abstract

**Background:**

Empathic erosion and burnout represent crises within medicine. Psychological training has been used to promote empathy and personal resilience, yet some training useful within adjacent fields remain unexplored, e.g., Stoic training. Given recent research within psychology suggesting that Stoic training increases emotional wellbeing, exploring this type of training within health professions education is important. We therefore asked: What impact would a Stoicism informed online training package have on third year medical students’ resilience and empathy?

**Methods:**

24 third year medical students took part in 12 days of online training (SeRenE), based on Stoic philosophy, and co-developed with psychotherapists. A mixed-methods study was conducted to evaluate impact. Pre- and post-SeRenE students completed the Stoic Attitudes and Behaviours Scale (SABS), Brief Resilience Scale (BRS) and Jefferson Scale of Empathy (JSE). All students completed semi-structured interviews following training and 2 months post-SeRenE. Thematic analysis was employed to analyse qualitative data, whilst within subjects t-tests and correlational analyses were conducted on quantitative data.

**Results:**

Quantitatively, stoic ideation, resilience and empathy increased post-training, with correlational analyses suggesting resilience and empathy increase in tandem. Qualitatively, four themes were identified: 1. Negative visualisation aids emotional and practical preparedness; 2. Stoic mindfulness encourages students to think about how they think and feel; 3. Stoic reflection develops the empathic imagination; and 4. Evaluating the accessibility of SeRenE.

**Conclusions:**

Our data lend support to the ability of Stoic-based psychological training to positively influence resilience and empathy. Although, quantitatively, results were mixed, qualitative data offers rich insight. The practice of negative visualisation, promoted by SeRenE, encourages student self-efficacy and planning, domains of resilience associated with academic success. Further, this study demonstrates a connection between Stoic practice and empathy, which manifests through development of the empathic imagination and a sense of empathic bravery.

**Supplementary Information:**

The online version contains supplementary material available at 10.1186/s12909-022-03391-x.

## Background

Empathic erosion and burnout represent two of the most concerning contemporary crises within medicine. Empathic erosion, defined as a loss of empathy, and burnout, a syndrome characterized by emotional exhaustion, cynicism and a low perception of accomplishment [1], are related - those exhibiting signs of burnout have less capacity to display empathy. Yet, training programmes which aim to cultivate empathy and personal resilience (a concept that protects against burnout) [2] are underdeveloped [3, 4]. There is an ongoing need for research into training within medical education that can combat rising levels of empathic erosion and burnout.

Empathy has been variously conceptualised within medical education, though scholars generally agree there are cognitive, affective and behavioural components to clinical empathy [5], and that empathy is important in improving health [6]. Halpern defines empathy as ‘an experiential way of grasping another’s emotional state’, distinguishing empathy from medicine’s long tradition of treating patients with an air of ‘detached concern’ [7]. Though there is debate regarding whether empathy decreases during medical school, a recent review of studies measuring Western medical student empathy surmised that the overall trend is for empathy to decline [8].

Burnout, ‘a state of mental and physical exhaustion related to work or care-giving activities’ is prevalent amongst students and, worryingly, is associated with suicidal ideation [1]. It is increasingly recognised that, in order to combat medicine’s burnout epidemic, both institutional-level and individual-level interventions are necessary [9]. Whilst institutional-level interventions commonly focus on improving working conditions, individual-level interventions most often focus on cultivating personal resilience amongst students and physicians. Both are important and work in complementary ways to address causes of burnout [9]. Personal resilience can be defined as an ‘emotional competence’ which involves the cognitive processes of self-efficacy, planning, self-control, commitment, and perseverance [10–12]. It has been discussed as an essential skill for doctors which can help mitigate against burnout [10, 13, 14] and empathic erosion [15, 16].

Yet, although the concepts of empathy and resilience are increasingly discussed, less is known regarding how curricula can most effectively encourage the development of these skills [3, 4]. Psychological training has been used to promote empathy and personal resilience within medical education- perhaps the most notable has been the rise in mindfulness-based interventions [17]. Yet, some forms of psychological training that have proven of use in cultivating empathy and resilience in adjacent fields remain unexplored within medicine- Stoic training (psychological training based on Stoicism, a philosophy of personal ethics), for example. Stoic training differs from the psychological training currently utilised within medicine and could offer different or complementary benefits to approaches that are more commonly used, such as mindfulness. Recent research within psychology suggests that Stoic training increases emotional wellbeing by decreasing worry and increasing participants’ senses of self-efficacy [18], which Bandura defines as a person’s beliefs concerning how well they can execute a certain plan in prospective situations [19]. Stoic training has, to the authors’ best knowledge, not been investigated within the context of medical education and offers an interesting interventional lens through which to study medical student empathy and resilience.

Stoic philosophy is widely misrepresented within medicine, portrayed as a negative ‘stiff upper lip’ that promotes detachment, suppression of emotions, and acceptance of extreme hardship with no motivation to challenge injustices in the world [20]. In actuality, Stoicism is an ancient Greek and Roman philosophy which offers a way to process the negative feelings one experiences whilst maintaining human connection [21]. Further, although Stoic skills and practices advocate for the acceptance of things that one cannot control, Stoicism does not promote inactivity in the face of systemic injustice. Though it is not a political theory, Stoicism is an ethic that encourages practitioners of the philosophy to act virtuously and become better people- within contemporary society, this must involve consideration as to how to engage in activism [22]. There has been a renewed interest in Stoicism within popular culture [23]- the Modern Stoicism movement has been conducting pilot studies since 2012 providing promising, yet not fully empirically validated, findings, regarding the association of Stoicism and Stoic training with well-being and resilience [24–26]. The Modern Stoicism movement has also been involved in developing Stoic Mindfulness and Resilience Training (SMRT), a comprehensive, intensive online Stoic skills training programme for the general public [27]. Stoic ideals underpin Cognitive Behavioural Therapy (CBT), though CBT has since evolved independently [28]. What is meant by psychological ‘Stoic training’ can differ between providers, but there are some Stoic practices foundational to the approach. These practices include: 1. Negative visualisation; 2. Stoic mindfulness; and 3. Stoic reflection. For those unfamiliar, these practices are summarised in Table 1.

**Table 1:** Description of core Stoic practices

Stoic practice	Description
Negative visualisation	Negative visualisation is a psychological practice in which participants visualise the bad things that could happen to them for example: what would happen if you were to lose your job, or if someone you cared about were to die? Negative visualisation as a term was first introduced within Stoicism by William Irvine [29], with ancient philosophers referring to this practice instead as ‘premeditatio malorum’ [30]. This exercise may appear pessimistic, but it is a powerful tool for developing appreciation for the things within your own life, and developing tangible plans for what you could do, were things to go wrong [29].
Stoic mindfulness	Stoic mindfulness concerns reflection on one’s own emotions and thoughts. Once this reflection fosters insight into an individual’s emotions and thought processes, individuals must actively remind themselves that emotions are projections of their own judgments- people assign labels to what they feel and experience which classify things positively or negatively. There are some things in life outside of our control- Stoicism advocates focusing your attention on only what you can control- often this is our emotional response to what happens to us or around us. Reframing negative emotions and experiences by exercising control over how you respond to what is happening can reduce rumination, catastrophising, pity and anger [30]. Stoic mindfulness differs from Eastern mindfulness-based practice- though both advocate careful reflection on one’s own attention, Eastern mindfulness attempts to root attention in the present moment, whereas Stoic mindfulness advocates for a focusing of attention on judgments and actions that we can directly control.
Stoic reflection	Stoic reflection is a type of daily reflection, where individuals look both forwards and backwards upon their day, rationally reflecting upon what is practically achievable, how they may approach setbacks, and the highs and lows of the day [31].

Research within psychology demonstrates that, as Stoic attitudes increase following engagement in the above practices, so do measures of emotional wellbeing and self-efficacy [18]. Given links between wellbeing and resilience [32], and the fact that self-efficacy represents one domain of personal resilience [10], Stoicism’s influence on resilience could be presupposed [33] but has not been proven within medical education. Further investigation is needed to demonstrate whether Stoic training influences resilience, and, if so, how. Regarding empathy, there is little empirical research connecting Stoic practice and empathy. There are interpretations of Stoicism which give short shrift to emotions and compassion that some scholars, such as Sherman, have argued is not representative of source texts [34]. Sherman advocates for ‘moderate Stoicism’, drawing on the writings of Seneca and Marcus Aurelius to highlight the central role of compassion to Stoic practice. Moderate stoicism stresses that the cultivation of empathy is critical, given humans’ innate connection to those around them [34]. Respect for each other (and within this, empathy for suffering) is the cement of society, and empathy is a pro-social behaviour [34]. Controlling one’s emotions within Stoicism is not about suppressing emotions, a popular misconception [35], but ensuring they are appropriate, and that people do not become emotionally enmeshed with one another whilst empathising [36]. However, despite such theorising, empirical exploration of the relationship between empathy and Stoic practice is wanting.

Given the relative dearth of research concerning interventions which facilitate empathy and personal resilience amongst medical students, and the unexplored possibility of Stoic training to assist in this plight, we asked: What is the impact of an online training package, based upon Stoic philosophy, on third year medical students’ resilience and empathy, initially and longitudinally?

## Methods

### Research approach

We employed a mixed method design and adopted a multi-paradigmatic stance to design and analysis. Multiple paradigm research selects different paradigms for qualitative and quantitative study elements [37]. We drew upon post-positivist epistemology and ontology for quantitative elements, and socio-constructivist ontology and interpretative epistemology for qualitative elements, in a ‘complementary strengths’ approach [37]. Within quantitative elements, we understand reality to be objective, but only ever imperfectly known due to the presence of human error- in regard to our study, we understand quantitative measurement of Stoic attitudes and behaviours, empathy and resilience to reflect an imperfect measure of the relationship between students’ experiences of the training package and the practice of empathy and resilience. Using a quantitative approach, we hope to say something generalisable about the relationships between the concepts of empathy, resilience and Stoic ideation. Within our qualitative arm, we understand empathy and resilience as personal, mental constructs, influenced by subjective social experiences. We draw upon qualitative research to investigate students’ subjective experiences of the training and explore the ways in which this education does, or could, support empathy and resilience. Within this research, mixed methods are important, as our qualitative findings add depth and context to our quantitative results, which we anticipated would be limited in terms of sample size and power, given this was designed as a pilot study.

### Online stoic training development

We have termed the online training students were asked to complete following voluntary enrolment in the study “*SeRenE*”- *Stoic rEflection for ResiliENce and Empathy*.

The training was developed by members of this research team (MB,AM,WL,GF,TL) using a Stoic training package created for a previous research project concerning the impact of Stoic training in a group of participants at risk of anxiety and depression, which AM developed [18]. We developed an additional exercise was developed to suit the purpose of this study as also interested in the cultivation of empathy (Exercise 3). Our combined expertise in psychotherapy (TL, AM), psychology (AM), and medical education (MB, WL, GF) meant we were well placed to develop such a training package. The developed training package was unique to this study, distinct from SMRT. Whereas SMRT lasts for 4 weeks, our training took place over 12 days. Further, SMRT includes exercises not included in SeRenE such as values clarification and worry postponement [38], and SeRenE includes materials not covered by SMRT, particularly exercises aimed at developing empathy.

The training read as a guided reflective diary, practically pitched, but rooted in Stoic Philosophy. It was designed to take 15–20 minutes each day for 12 consecutive days. An overview of SeRenE training is offered (Table 2), with the package provided in full (Additional file 1).

**Table 2:** Description of SeRenE training package exercises

Training exercise	Description
Exercise 1: Predicting Misfortune	Promotes practice of negative visualisation. This exercise asked students to predict what they might find difficult, challenging, or what could go wrong in the day ahead. It promoted planning for what could go wrong and encouraged reflection as to how students might deal with negative turns of events.
Exercise 2: Examining Judgments	A core principle within this exercise is the assumption that we have a certain degree of control over the way we feel, and that it is our interpretation of events around us that make them good or bad. This exercise promotes the practice of Stoic mindfulness. Students were asked to note down some of their impressions or judgments from the previous day, or the current day, and examine each of the judgments that have made in turn that have led to the way they have interpreted events in their life.
Exercise 3: Developing Empathic Reserves	This was an additional exercise developed to suit the purpose of this study as also interested in the cultivation of empathy. This exercise was developed in consultation with two qualified psychotherapists (AM and TL). Exercise 3 was a more targeted and specific version of exercise 1. Students were asked to consider a situation where they may need to offer empathy to a patient, think about what could go wrong and how they could prepare for the possibility of such issues. We hoped this exercise would challenge students’ negative experiences of empathy and promote emotional preparedness.
Exercise 4: Evening Reflection	The aim of this exercise was to promote Stoic reflection. Ideally, this exercise would be completed at the end of the day, summing up the thoughts and actions of the day. The focus was on what had been unhelpful, what was left undone that students wanted to do, and a list of things done well. We hoped this exercise would promote honest appraisal of thoughts and actions and offer a chance to prepare for troublesome of problematic ways of thinking the next day.

### Participants

As ‘the devil is in the third year’ regarding empathy loss amongst students [39], we recruited third year medical students at the Hull York Medical School (HYMS) as a convenience sample by email in the academic year 2020–21 by email. We offered participants a £20 Amazon voucher in recognition of the significant time they would spend completing training and follow up.

We recruited 25 students, with a final sample of 24 completing SeRenE. We sent participants links via institutional emails inviting them to complete a new day of SeRenE at the start of every working day, for 12 days. SeRenE was hosted in EasyGenerator. It was deemed acceptable to miss a maximum of 2 days of SeRenE for data to be eligible for analysis, with three students doing so. On average, students spent 23 minutes engaging with SeRenE daily.

All of our recruited participants were enrolled in SeRenE. We considered a control group of students, which would have allowed us to compare and contrast experiences, but unfortunately did not have the resources to also run and evaluate a control group. Future research should consider the use of a control group in the resource-planning phase of project design.

### Impact of Covid-19

In March 2020, Covid-19 disrupted medical education. At HYMS, third years’ clinical placements were suspended, following 6 months of full-time placement, and students shifted to online learning. Students commenced SeRenE from May 2020 onwards, thus completing training and interviews whilst clinical placements were suspended. Some students chose to volunteer to assist with the pandemic effort in clinical or non-clinical roles, but this was non-mandatory.

### Ethics

We obtained ethical approval from HYMS (approval number: 20 04). Once written, informed consent was obtained from participants, we enrolled them in SeRenE.

### Quantitative data collection

Prior to training, we asked participants to complete the Stoic Attitudes and Behaviours Scale 5.0 (SABS), Brief Resilience Scale (BRS) and Jefferson Scale of Empathy, student version (JSE-S). We requested participants complete these measures again post-training.

The BRS presents as 5-point Likert type scale with 6 items and measures ability to bounce back after difficulty [40]. In both the initial development of the scale [40] and subsequent cross-cultural validation studies (see [41] for a review) the BRS has displayed reliability and construct validity for measuring resilience as a unidimensional construct, namely the ability to bounce back. Possible scores range from 1 (which represents low self-rated resilience) to 5 (high self-rated resilience). Pre-training, the BRS achieved a Cronbach’s alpha of 0.823, indicating a high level of internal consistency, and has been utilised in previous research involving medical students [42]. The JSE is well-used within healthcare trainees and presents as a 7-point Likert type scale with 20 items as a measure of self-reported levels of empathy. The JSE has displayed strong construct validity in measures of empathic concern, warmth and dutifulness and perspective taking [43] as well as consistent significant associations between JSE scores and patient rated physician empathic communication [44, 45]. An acceptable internal consistency was achieved pre-training, with a Cronbach’s alpha of 0.724. The SABS presents as a 7-point Likert type scale with 60 items [25]. Participants are asked to rate statements on a scale of Strongly Agree (7) to Strongly Disagree (1) [24]. SABS generates a score between 60 and 240, with higher scores indicating higher levels of Stoic ideation [25]. The SABS scale, whilst still nascent as a measure of Stoic ideation, demonstrated a high level of internal consistency with a pre-training Cronbach’s alpha value of 0.867, and so was deemed appropriate for use.

### Qualitative data collection

All 24 students also completed in-depth, semi-structured interviews via video-conferencing software immediately following training with either MB or WL concerning their experiences of the training package and thoughts regarding empathy and resilience. After 2 months, we (MB or WL) re-contacted participants for a follow-up virtual semi-structured interview concerning the impact of SeRenE. We considered longer term follow up but were constrained by delivering results on a grant timeline, and so selected 2 months as our point for longitudinal evaluation. All 24 students completed follow up interviews. Interview duration ranged from 21 minutes to 58 minutes. Semi-structured question stems are available (Additional file 2). A professional transcriber transcribed interview audio verbatim and anonymised data for analysis.

### Quantitative analysis

We conducted statistical analyses on all self-report scales pre- and post-training using SPSS®. We conducted within subjects t-tests to assess self-report score change pre- to post-training and correlational analyses to assess the relationship between variable change.

### Qualitative analysis

Four researchers (MB,WL, UO, GH) analysed all interview data inductively using Braun and Clarke’s reflexive approach to thematic analysis [46]. They followed six steps: 1. Familiarising self with data; 2. Generating initial codes; 3. Searching for themes 4. Reviewing themes; 5. Defining and naming themes; 6. Producing a report. All authors read and re-read study transcripts to familiarise themselves with the data. Descriptive initial coding was undertaken by UO and GH in NVivo, with MB and WL acting as second coders to deepen analysis. All four authors met to discuss initial coding, and a codebook was created and systematically applied to all data. The authors then met again to discuss early themes. MB reviewed all themes and refined the codes within each. Themes were named and defined following group discussion. MB created a narrative report and circulated this to the research team for discussion.

We theoretically situated study findings by using background literature abductively to inform data analysis at the defining and naming themes stage. In this way, our thematic analysis was informed by ‘sensitising concepts’ [47, 48]. The sensitising concepts that informed analysis are the conceptualisations of empathy, resilience and Stoicism outlined within the conceptual framework of our introduction. This approach has been described by Varpio et al. as ‘theory-informing inductive data analysis’ [49].

We utilised reflexive journals to explore researcher assumptions and beliefs. MB, WL and GF have previously researched empathy within medicine, focusing largely on students’ struggles. AM is a psychology PhD student, interested in Stoic training. TL is a psychotherapist who draws upon Stoic philosophy in practice. GH is a third-year medical student, a member of the community under study. UO is a medical education PhD student. Given the active roles MB, AM, WL and TL had in developing this training package, the authors critically reflected on their experiences and assumptions, challenging positive interpretations in particular with the help of authors not involved in training design (GF,UO,GH).

## Results

### Demographics

All 24 participants were third year medical students. Demographic data is available in Table 3.

**Table 3:** Demographic data

Age	Gender	Ethnicity	Sexual orientation
Range: 20–27 Mean: 21	F 15 M 9	White British: 12 Chinese: 2 Any other mixed/multiple ethnic background: 3 Black British: 2 Any other white background: 2 Black African: 1 Asian British: 1 White Irish: 1	Heterosexual: 19 Gay: 3 Bisexual: 2

### Quantitative analysis

Means and standard deviations of self-report scores are presented in Table 4. Paired-samples t-tests were conducted to compare pre- to post-training scores on the self-report measures. Stoic ideation increased after training, t (21) = 3.009, *p* = 0.007, d = 0.641; as did Resilience, t (23) = 2.469, *p* = 0.021, d = 0.504; and Empathy, t (23) = 4.848, *p* < 0.001, d = 0.990. A post-hoc G* Power Analysis [50] revealed a power of 0.905.

**Table 4:** Means and standard deviations (in parentheses) for self-report measures pre-training, post-training and mean change in score

Measure		Mean Score	Minimum Score	Maximum Score
Stoic Ideation	Pre-Training	277.77 (27.53)	227.00	343
(SABS)	Post-Training	297.82 (33.56)	211.00	350
	Δ SABS	20.05 (31.25)	− 51.00	92.00
Resilience	Pre-Training	3.19 (0.90)	1.83	5.00
(BRS)	Post-Training	3.63 (0.84)	2.16	5.00
	Δ BRS	0.44 (0.88)	−1.01	2.40
Empathy	Pre-Training	99.46 (19.09)	67.00	128.00
(JSE)	Post-Training	122.46 (12.87)	92.00	138.00
	Δ JES	23.00 (23.24)	−18.00	64.00

Correlational analyses of the change in self-report measures were conducted, finding a significant positive correlation between BRS score change and JSE score change, r (24) = 0.480, *p* = 0.018, 95% CI 0.071, 0.751], suggesting that as resilience increases so does empathy. There was no significant correlation between SABS score change and BRS score change r (22) = 0.282, *p* = 0.204, 95% CI [−0.167,0.634]; or JSE score change, r (22) = 0.240, *p* = 0.282, 95% CI [− 0.208,0.605], suggesting no relationship between Stoic ideation change and resilience or empathy change. A post-hoc G*Power Analysis [50] revealed a power of 0.687.

### Qualitative analysis

Analysis generated four themes: 1. Negative visualisation aids emotional and practical preparedness; 2. Stoic mindfulness encourages students to think about how they think and feel; 3. Stoic reflection develops the empathic imagination; and 4. Accessibility of SeRenE.

Student quotes are labelled using participant and interview numbers.

#### Negative visualisation aids emotional and practical preparedness

When discussing their experiences of SeRenE, students stressed the utility of the exercises which facilitated negative visualisation (Exercises 1 and 3). This sort of reflection helped students to consider what might go wrong in a variety of situations and, in doing so, they were able to generate contingency plans to deal with outcomes different than what they hoped or expected. This was an element of the training that had impact at 2 months.

“One of the questions was asking about what am I going to do, and what could go wrong and how would I deal with it. So I do plan now, and I do think about how to … what to do and how to restructure things when stuff can’t happen at the right time or stuff like that.”

Student 15, Interview 2

Thinking in this way improved student confidence in clinical situations, as the fear of something going wrong was lessened.

“With the wards sometimes I feel anxious not knowing many people and still being quite new...but if I step back before I walk on and just sort of plan in my head what I want to do and what I would do if that doesn’t work out, I feel less uncertain and also less worried about saying the wrong thing or doing the wrong thing. That sort of thought process gives me confidence.”

Student 23, Interview 2

Though some may assume that negative visualisation would promote pessimism, students felt such exercises decreased the chance of them catastrophising when things did go wrong. Further, students identified negative visualisation as a more positive and proactive approach to reflection than models of reflection they had previously experienced. By thinking of, and orientating oneself to, solutions, students felt able to focus on problem-solving, as opposed to ruminating on what had happened.

“I feel like it’s allowed myself to sort of think about a more positive or beneficial way of things going … that’s what I particularly liked about the whole process.”

Student 12, Interview 1

The emotional preparedness negative visualisation fostered was perceived by students as increasing personal resilience by reducing stress and promoting adaptability in the face of change.

“It gives you more resilience because you can anticipate stuff going wrong. You can prepare yourself … and also give yourself some time to think about what’s already happened,”

Student 16, Interview 1

Additionally, students felt their productivity, both on and off the wards, increased as a result of practicing negative visualisation. In thinking about what could go wrong, students more readily had backup or contingency plans which acted as a sort of practical preparedness.

“When I’ve been at work in the hospital if something has gone wrong, because I’ve already thought through what might happen to myself when I start out on that task, if something does happen then I can just change my approach and what I’m doing really quickly … which is more efficient.”

Student 22, Interview 2

#### Stoic mindfulness encourages students to think about how they think and feel

It was evident that engaging in a Stoic sort of mindfulness encouraged students to reflect on their own emotions and responses to different situations. This was achieved, at least in part, by focusing attention on the present day, and what was happening around students in the ‘daily reflection’ exercise.

“I think it’s helped because I’ve never had any exercises where I had to reflect on my day and I think it’s helped in terms of me sort of understanding my feelings and why I feel a certain way.”

Student 3, Interview 1

Alongside an increased awareness of their own emotional state, SeRenE also facilitated consideration, within some students, of *how* they were thinking. In technical terms, Stoic mindfulness promoted metacognition.

“I was quite surprised actually that it did make me think differently. After a couple of days I did sort of … I was taking note more of my thought processes and my actions and how I was viewing things … I was like, “okay today I need to think in a certain way.” So that … was a really positive thing … it’s made me change the way I think about situations.”

Student 12, Interview 1

Such metacognition helped some students to challenge what they thought, and several students voiced thinking differently about situations that had previously made them angry or upset because of their engagement in SeRenE.

“It’s actually realising when you are having judgments, like actually recording throughout the day and then realising it’s not a situation you can control, but you can control your responses. That was brilliant. Definitely very useful.”

Student 19, Interview 1

Other students voiced that an increased awareness of their emotional state, thought processes and achievements helped them to recognise when they were being overly pejorative with themselves and fostered a kindness towards themselves.

“The last section that was like … what did you do well, what didn’t you do well, I think that was quite useful to sort of say, well I’ve done well today. I don’t have to completely beat myself up about it, it’s not all gone wrong”.

Student 7, Interview 1

Some students admitted to continuing to use SeRenE training formally and within written reflections 2 months after the training was completed in their follow up interviews. For others, the changes in thought processes and practices were more subconscious.

“Subconsciously I do reflect more. I haven’t done as much formally like writing it down I did during the training but definitely I catch myself more doing it at the end of the day right before bed.”

Student 3, Interview 2

#### Stoic reflection develops the empathic imagination

Within SeRenE, exercises which promoted Stoic reflection (exercises 3 and 4) promoted a more empathic approach to patients and clinical care. Students found that reflecting on patient cases and what could go wrong helped them to consider the patient perspective.

“It helped me look at things from the patient’s point of view … I think it will help me understand patients a bit better as I now put myself in their shoes more.”

Student 3, Interview 1

Considering why a situation involving empathy could escalate or derail helped students to consider patient context and, interestingly, socioeconomic determinants of health that may be at play within a consultation.

“It has been interesting to think about what could go wrong because sometimes I’m thinking “Well, what’s happening with this person at home?”. Is there something going on in the home like a relationship breakdown or is someone they love suffering. And that thought process gives me more empathy for them, because you never really know how hard someone’s life might be and how that might make them angry or lash out with behaviours like drug use.”

Student 24, Interview 2

In addition, students felt more able to put themselves in their patient’s shoes as a result of engaging in reflection regarding empathy. This seemed borne of a sensitivity to students’ own judgements and feelings- with this sensitivity came an appreciation for others’ feelings and judgments, too.

“Evaluating our own judgement and our own feelings towards things can be helpful to like remind us when we’re in a clinical setting to take into account the kind of things that patients may be judging and feeling themselves. So in that case it helped with putting yourself in their kind of shoes.”

Student 2, Interview 1

Some students felt able to mobilise these empathic insights to make changes to their practice …

“The patient scenario, it does make me think twice about what I say and do and what my body language is showing to the patient because that will definitely influence the consultation”.

Student 4, Interview 1

… but for others, not knowing how to change their practice in regard to these new insights generated uncertainty. There were suggestions that SeRenE could be incorporated into communication skills training, where students could reflect on these insights as a group and work with tutors to amass new skills for situations they had identified as potentially troublesome.

“ … with the patient thing, the last question I think was how would you cope with what went wrong? I think maybe because of the way I think, I feel like I was giving the same answer every day … I would maybe need contact with mentors to get more practice on how I would better myself for the future if a similar consultation happened.”

Student 8, Interview 1

#### Accessibility of SeRenE

The final theme within our data concerns the accessibility of SeRenE - what worked in practice, and what did not.

The training was felt to be easy to use.

“It was really simple to use and easy log-in and it was really helpful to have the hints.”

Student 10, Interview 1

The most commonly voiced difficulty in regard to the accessibility of SeRenE concerned exercise 2, ‘Evaluating Judgments’. Several students misunderstood what was meant by the term ‘judgments’, taking it to instead mean ‘judgmental’, which hampered engagement.

“I guess it’s just the kind of person I am, but I don’t usually judge people”

Student 4, Interview 1

Several students noted the utility of the examples provided on the first day of training and would have liked to have been able to access these worked examples every day. For some students, particularly those who did not volunteer during Covid, patient cases were difficult to think of within exercise 3, and so additional examples to prompt reflection were desired.

“I found it difficult to think of a different clinical scenario each day … because we’re not on clinical placement at the moment.”

Student 20, Interview 1

There was discussion within students’ follow up interviews regarding the necessary length of SeRenE, and the need for repetition over time. Whilst, generally, students felt the initial training was of appropriate length, there was disagreement regarding whether it would be beneficial to repeat training during an academic year and, if so, how frequently. What became apparent was that student preferences differed, and a flexible approach following initial training, where students can reengage at their own leisure, may serve the needs of more students than a mandated approach. Though the training platform was well received, having to access SeRenE through a web browser was off-putting for some. Student preference seemed to be for an app that could be accessed on-the-go.

“I really loved how it was spread out over 2 weeks because it actually meant you had a lot of time to process it and then you could think from one day onto the next.”

Student 6, Interview 1

## Discussion

We investigated the impact of a training package (SeRenE) based on Stoic philosophy on medical student empathy and resilience. Both quantitatively and qualitatively, there is evidence SeRenE was effective in supporting the development of empathy and resilience, and areas of the training that would benefit from further development.

Quantitatively, the results from self-report measures are mixed. Self-report scores all significantly increased post-training, consistent with previous literature [18]. The correlational analyses indicate a positive relationship between the change in resilience and the change in empathy. This may support previous literature suggesting that improving resilience is a viable mechanism for protecting against empathic erosion [15]. However, the lack of a significant relationship between Stoic ideation, resilience and empathy raises concerns. Previous research investigating Stoic training has found significant positive quantitative effects of Stoicism on self-efficacy [18], a factor related to empathy and resilience in healthcare [51] and other professions [52]- opening an avenue for future study.

Qualitatively, there is more robust evidence that SeRenE positively influenced student empathy and resilience. The exercises within SeRenE that promoted negative visualisation (exercises 1 and 4), were received particularly positively by students. Students felt engaging in negative visualisation promoted emotional and practical preparedness that bolstered personal resilience. Thinking forwards and preparing for what could go wrong engaged students in planning and fostered a sense of self-control and self-efficacy, core domains of personal resilience [10–12]. In particular, self-efficacy, which Bandura defines as ‘self-belief about how well one can execute courses of action required to deal with prospective situations’ [19] seemed to increase as a result of engagement in training, suggesting that negative visualisation prepares students for future situations. This supports previous Stoic training research, which suggests that wellbeing increases following engagement through the mechanism of self-efficacy [18]. Given that higher levels of self-efficacy are associated not only with personal resilience, but also with academic success [53] and motivation [54], this is an important novel finding in the context of medical education. Further, other types of psychological training currently employed within medical education do less to encourage self-efficacy and planning. Mindfulness, for example, focuses attention on the present moment and so, although of evidenced use [17], does less to nurture the domains of personal resilience encouraged by Stoic training. Given the importance of self-efficacy to wellbeing, interventions which encourage development of this skill are also of value.

In addition to the benefits associated with the practice of negative visualisation within SeRenE, this study’s qualitative results indicate the ability of the training to forefront considerations of empathy amongst students. There has long been a concern within medical education that doctors are encouraged to manifest and practice ‘detached concern’– a type of empathy where clinicians distance themselves from patients so that, although they may be saying empathic things, they do not truly *feel* empathy for that person and are empathizing on a purely cognitive level [55]. Contemporary definitions of clinical empathy within medical education acknowledge that empathy involves cognitive, affective and behavioural components [5]– it cannot be cognitive alone, as such empathy can feel ‘fake’ or forced [56], and lead to the distressing phenomenon of ‘empathic dissonance’, where pressure is felt to deliver empathy cognitively, when none is felt affectively [57]. Stoicism has a bad reputation in many circles in regard to empathy- indeed, there are interpretations which maintain that there is no role for empathy in a Stoic [58]. Thankfully, other interpretations exist which acknowledge the importance of empathy in promoting citizenship and normalise the experience of emotions [34]. This research suggests that, through the practice of negative visualisation and daily reflection, students *can* develop their empathic imaginations, more readily feeling able to put themselves in their patients’ shoes. What we witnessed was not the promotion of detached concern but, instead, recognition of one’s emotional state and work to make those feelings manageable, rather than suppressing them. In this way, SeRenE promotes empathy both through offering the time and space to consider patients’ complex lived experiences, but also through fostering a sort of empathic bravery – a willingness and eagerness to engage in empathy with patients, given students felt more prepared for a variety of outcomes resulting from them offering affective empathy to patients. There were some ways, however, in which this benefit could have been developed further. Some students found it difficult to think of solutions to prospective empathy situations they had little-to-no experience of. Whilst we are likely all able to think of alternative plans for aspects of our personal life (such as what we would do if our plan to go to the gym was derailed), it seemed harder for medical students with limited clinical exposure to consider alternative plans for if things went wrong in regard to empathising with patients. There is scope to integrate SeRenE alongside established communication or clinical skills modules within curricula. Such integration would offer both a private space to reflect on practice and a public forum to seek advice as to possible solutions.

It is important to consider other ways in which SeRenE could evolve to better serve the needs of students. The recommendations made by students, and those we have generated from our analysis, are summarised in Table 5.

**Table 5:** Recommendations for future iterations of SeRenE

Issue	Recommendation
The term ‘judgments’ within exercise 2 was confusing for some students. This led to misinterpretation of what this exercise was asking for amongst some, and lessened the potential impact of this exercise, which was intended to promote Stoic mindfulness regarding what can, and cannot, be controlled.	Reconsider use of the term ‘judgments’. Although this is a technical term associated with Stoic Philosophy, it was widely misunderstood. We suggest that the alternative term ‘interpretations’ is trialled to encourage students to consider the negative or positive labels they assign to emotions or experiences. Be open to re-evaluating the effectiveness of this exercise following modifications to the language used.
Some students found it difficult to consider possible solutions to the practice of negative visualisation in regard to empathy, given limited clinical exposure.	Integrate SeRenE alongside established communication skills/clinical skills modules within health professions training or ensure delivery of an appropriate in-person follow up, where students have a chance to discuss possible solutions with peers and senior clinicians. This may also increase engagement.
It could be difficult to remember exactly how to complete each exercise each day, as the worked examples were only visible on day 1 of the training.	Provide access to the worked examples on each day of training.
For students who were shielding, or did not volunteer clinically, a lack of patient contact made considering patient cases to reflect on more difficult.	This training is most appropriate for students experiencing regular patient contact. If it is used in situations where patient contact is limited in the future, a bank of clinical examples should be offered for students to reflect on.
There was no consensus regarding whether training should repeat within an academic year, and how frequently this should be done.	A flexible approach following initial training may be most suitable, where students can, and are encouraged to, re-engage at their own leisure.
Having to access training through a web browser was off-putting for some.	An app could be developed to host SeRenE, which would increase the accessibility of, and possibly the engagement, with such training in future.

### Limitations

There are three clear quantitative limitations within this study: a small sample size, as confirmed by the low power achieved; uncertainty regarding scale utility; and the risk of self-selection bias. A low sample size is a common problem in psychology experiments, which can cast doubt on the effects identified [55]. To confidently detect effects in this type of correlational analysis, the study should be completed by a minimum of 31 participants, according to a G*Power analysis [50]. Unfortunately, recruitment of this number of participants was not possible. This should be considered when interpreting results.

Additionally, there is uncertainty regarding the utility of some of the scales used – all scales rely on self-reports, which is potentially questionable [59, 60]. Though our conceptualisation of empathy includes cognitive, affective, and behavioural domains, Hojat’s conceptualisation of empathy within the JSE is largely cognitive [61]. Whilst SABS measures Stoic ideation across many domains, our training focused only on three Stoic practices, meaning that SABS, whilst the best-reputed scale in regard to Stoic practice, was not completely suited to measuring SeRenE’s impact.

Self-selection bias is a common risk in research utilising voluntary, purposive sampling strategies. Those interested in this work must consider that the students who volunteered to participate in this research may have already been interested in Stoicism, empathy, or resilience, which could skew results and increase the perceived positive impact of SeRenE. Future research considering the impact of a mandatory SeRenE training package would add depth to our early positive findings.

In more general terms, the educational impact of Covid-19 is somewhat limiting in terms of reduced student-patient interactions during the study. As outlined within the discussion, SeRenE is most appropriate for students regularly interacting with patients. The results of this study may be under-emphasized, given reduced levels of patient contact during Covid-19. Further, the impact of Covid-19 on student empathy and resilience in and of itself remains unclear. As data are gathered by others to illuminate the impact of Covid-19, the results of this study should be reconsidered in light of any new evidence.

## Conclusion

In conclusion, our data lend support to the ability of psychological training based on Stoic philosophy to positively influence student perceptions and experiences of resilience and empathy. Although quantitatively, results were mixed, the quantitative arm was underpowered, and this data must be interpreted alongside the rich context offered qualitatively. In sum, we have identified that the practice of negative visualisation, promoted by SeRenE, encourages student self-efficacy and planning, domains of personal resilience that are associated with academic success and motivation. Further, this study makes apparent the connection between Stoic practice and empathy for patients, which manifests through development of the empathic imagination and a sense of empathic bravery garnered by increasing levels of self-efficacy. These are important novel findings, as they emphasize the need for further large-scale research concerning the role of Stoic training in promoting self-efficacy. Qualitatively, longitudinal investigation of the impact of SeRenE with different stages and types of health trainees holds merit and would cast further light on the mechanism and impact of Stoic training within healthcare.

## Supplementary Information

**Additional file 1.**

**Additional file 2.**

## Data Availability

Consent was not sought from participants to share data beyond the listed research team, and so data is not publicly available. Please contact the corresponding author of this article if you would like to request data.

## Abbreviations

SMRT: Stoic Mindfulness and Resilience Training
CBT: Cognitive Behavioural Therapy
HYMS: Hull York Medical School
SeRenE: Stoic rEflection for ResiliENce and Empathy
SABS: Stoic Attitudes and Behaviours Scale 5.0
BRS: Brief Resilience Scale
JSE: Jefferson Scale of Empathy
MB: Megan Brown
AM: Alexander McLellan
WL: William Laughey
GF: Gabrielle Finn
TL: Tim LeBon
UO: Usmaan Omer
GH: Ghita Himmi
