# Supplementary material for: Can stoic training develop medical student empathy and resilience? A mixed-methods study
Source: BMC Med Educ. 2022 May 3;22:340. doi: 10.1186/s12909-022-03391-x (PMC9064267; doi:10.1186/s12909-022-03391-x)
Supplement: Supplementary file 1 — Additional file 1. [file 12909_2022_3391_MOESM1_ESM.docx]

**Additional file 1**

**SeRenE- Stoic rEflection for ResiliENce and Empathy- Training package**

**Session 1 - Screen One**

Welcome to Session 1 of the training. This day will provide you with an overview of what you can expect for the next 12 sessions, and your first training material.

If you have any questions during this training period, please email the research team and we will be happy to help.

**Session 1 - Screen Two**

You will be asked to completed four exercises every day.

These exercises can be completed at any time of the day, though recommendations are made at the beginning of each exercise and instructions will be presented each time you are due to complete the exercise.

Please write as fully with as much detail as you can. Your answers are stored securely and are anonymised.

**Session 1 - Screen Three**

**Exercise One: Predicting Misfortune.**

The first exercise is to predict what you will find difficult, challenging or what could go wrong in the day ahead.

What is the purpose of beginning our days with a thought process like this? Much negative emotion and experience comes from being met with an unexpected negative outcome. We are let down when our friend is late or get angry with someone being rude to us. We get anxious when we are uncertain of how to act or when our desired outcome looks unlikely. By predicting the difficulties we are to face that day, we are able to take a proactive approach and be prepared.

The purpose of this exercise is not to promote pessimism, but to promote preparedness. If we are anxious about going to an interview, we should mentally prepare for being anxious in the interview, and ask ourselves how we may deal with it. When we begin the day having prepared four our displeasure at being on a cramped train, or our disappointment at getting a poor grade on an essay, we are better prepared to handle the negative situation should it arise. We do not purchase phone insurance in the hopes that we lose our mobile phone, but to protect and support us should we meet with misfortune. This exercise is your insurance policy should the day turn out to be a bad one, protecting ourselves from the worst of the negative emotional experience should it come to pass.

**Session 1 - Screen Four**

***Instructions:***

Each day you will be asked to predict what misfortune you may meet either:

- For the rest of the day if you are completing this in the morning.
- For the next day if you are completing this in the afternoon/evening.

If you are completing this in the afternoon for the next day, it is highly recommended you read this list over or complete it again in the morning, as this is the preferred time period to complete this exercise in.

To complete this exercise:

1. List the activities or actions you will be taking part in that day as well as things you want to achieve. For example:

- Going to the gym
- Attending classes/work
- Get positive feedback on a project
- Have a fun evening out with friends

2) Think of and write out what misfortune may befall you with regard to each of these items, and how you would cope with it. For example:

- Going to the gym. **I may not have time to go to the gym, or I may not feel like it.**
- Attending classes/work. **I may feel frustrated that I can’t understand a lesson/complete a task. My teacher/manager may annoy me or say something that knocks my confidence and make me doubt myself**.
- Get positive feedback on a project. **I may not get the positive feedback I hoped for.**
- Have a fun evening out with friends. **I may be kept late due to work commitments and not meet them when I want, which may affect my mood negatively. My friends may cancel on me or forget to show up. We may not have a fun time when we are out for several different reasons.**

3) Think of and write out what you would do to cope with if each misfortune where to happen. For example:

- Going to the gym. I may not have time to go to the gym, or I may not feel like it. **If I do not have time, I will attempt to go for a shorter period of time to fit it in or go another day. If I do not feel like it, I will attempt to go for a shorter period of time just to keep my routine going.**
- Attending classes/work. I may feel frustrated that I can’t understand a lesson/complete a task. My teacher/manager may annoy me or say something that knocks my confidence and make me doubt myself. **I can deal with this by reminding myself they are not in control of how I feel. If I do not understand or cannot do something, I will ask person XYZ for help.**
- Get positive feedback on a project. I may not get the positive feedback I hoped for. **If this happens, I will remember that another person’s opinion on my work is out of my control and I can ask for detailed feedback to make sure I am able to better understand and meet their standards.**
- Have a fun evening out with friends. I may be kept late due to work commitments and not meet them when I want, which may affect my mood negatively. My friends may cancel on me or forget to show up. We may not have a fun time when we are out for several different reasons. **If I am running late, I will let them know so I don’t feel bad, and will try to not let it affect my enjoyment of the night. If my friends cancel or do not show up, I will do ‘XYZ’ instead. If we do not have fun for whatever reason, I recognise that those reasons are out of my control and will let it go.**

**Session 1 - Screen Five**

**Exercise Two: Examine Our Judgements.**

A core principle here is the assumption that we have a certain degree of control over the way we feel, that it is our interpretation of events around us that make them good or bad. A helpful thought when faced with challenges is that these obstacles are simply facts of the world rather than offences against *us*. This being the case, they would not need further interpretation. They do not need to mean anything about us or for us, they simply *are* and can therefore be dealt with.

Another way of describing this principle is to take a view from above. When certain events can seem to be all consuming and awful, if we were to take a bird’s eye view, we may be able to view them as less permanent, gain a different perspective and make them seem more manageable.

**Session 1 - Screen Six**

***Instructions:***

Each day you will be asked to note down some of your impressions or judgements from:

- The previous day if you are completing this in the morning.
- For the current day if you are completing this in the afternoon/evening.

This exercise is best completed in the evening when things are still fresh, or you can keep a record of your judgements on your phone or in a book throughout the day for examination later. To complete this exercise:

1. Write down a list of the judgments you have made, for example:

- Person X was very rude
- Situation Y made me anxious
- Event Z means I am going to have a hard time tomorrow
- Having the new Phone Z would make me happier

1. Examine each judgement in terms of the sequence of events that led to that interpretation. For example:

- Person X was very rude. **Person X did not say much and seemed unenthusiastic when I greeted them with a smile and friendly words this morning. I felt they should have been enthusiastic back.**
- Situation Y made me anxious. **I was uncertain of what to do heading into Situation Y. Many people were looking at me. I felt pressure and then became anxious.**
- Having the new Phone Z would make me happier. **I saw and advert for the new phone. I have seen many adverts for the new phone which make it look attractive. I have spoken with other people about this phone. I imagined myself with the new phone and other people asking me about it. I felt desire for the new phone.**

Session 1- Screen Seven

Exercise Three: Developing empathic reserves

The aim of this exercise is to reflect upon your empathy for patients and the emotional response this can sometimes provoke. Similarly to exercise one, you will engage in a process of ‘predicting misfortune’, this time specifically in regards to patient situations necessitating empathy. Offering empathy to patients can be difficult. You may feel it is difficult to control your emotions, you may feel the best way to handle distressing situations is to mentally detach from them and this may impact the type of empathy you give. This exercise will aim to prepare you for situations where you may feel distressed offering empathy. By visualising difficult situations in this guided way, we hope you will feel more prepared to deal with these situations when they arise in the future. Not only will this exercise then act as a proactive tool you can use to protect yourself from an extreme emotional experience, but it will allow you to engage in a genuinely empathic way with patients, free of this fear.

Session 1- Screen Eight

Each day you will be asked to think about a situation where you may need to offer empathy to a patient. Try to think of a different patient situation each time. These scenarios can be based upon real-life patients you’ve met, or real-life situations you found emotionally difficult. You will then be encouraged to visualise this situation as distressing to you personally, for whatever reason. Finally, you will be guided to visualise yourself coping well with the situation and offering patients genuine empathy.

To complete this exercise:

- 1. Consider a situation where you may need to offer empathy to a patient. This could be a situation you have personal experience of, or a completely imagined scenario. This exercise will work best if this situation is one that holds the potential to prove emotional. Write a brief description of this scenario and how you may offer empathy below. **For example, you may be on a palliative care rotation and speak to a patient with terminal cancer.**
  2. Now imagine this situation does prove emotional. Imagine feeling distressed during the patient encounter. Consider how you could feel and why, and jot down some thoughts below. **I may feel upset as the patient is close to me in age. I may feel distressed hearing about the intensity of their struggle. We may share a hobby, for example cycling, which the patient can no longer do and is upset about.**
  3. Think about what could go wrong if you become distressed or emotional. List these possibilities below. **If I become emotional, I may cry and make a fool out of myself in front of the patient and consultant. If I become distressed, I won’t be able to focus and take a good history. Etc.**
  4. Step back and now instead imagine the same situation going well. You do not feel distressed, even though you offered the same empathy. Is there anything that you did differently or any thoughts you had that were different this time? If so, list these below. **Instead of focusing on the intensity and difficulty of the patient’s struggle, I focused on how inspiring their perseverance and bravery was. When considering our shared hobby and closeness in age, I tried to focus on feeling thankful for my own fortunate set of circumstances.**
  5. Look back on your thoughts regarding what could go wrong and challenge these. As empathy involves imagining situations from the patient’s perspective, you may feel some of what the patient is feeling- sadness, anger, fear. It is okay to feel this way- it is how we move past these initial feelings that allows us to cope, whilst still understanding that patient’s perspective. Understanding a patient’s emotions helps us make better patient-centred decisions. Try to remember that you only have control of your own actions and are not in control of the outcome to those actions. As these outcomes, including the patient’s emotional response, are beyond your control, try not to dwell or ruminate on what happens. Ruminating takes away your energy from other things you could be doing and is damaging in the long run. So long as you were genuinely empathic and engaged with your patient, you were being an excellent caregiver. Re-list your thoughts from question 3 below and challenge these, in line with the advice offered above. **‘If I become emotional, I may cry and make a fool out of myself in front of the patient and consultant’ 🡪 I can try to minimise the chance of this happening by emotionally preparing for this situation. If this does happen, I can remember it is natural to feel this way and that I have no control over the patient and consultant’s response to my actions. As such, I should not dwell on what has already happened in this scenario, and instead focus on emotionally preparing myself for future consultations. ‘If I become distressed, I won’t be able to focus and take a good history’ 🡪 emotionally preparing for distressing situations using this exercise will help control your own actions and so maintain focus. In the situation itself, allow yourself to feel the patient’s emotions, sit with them for a moment, then try to move past ruminating upon these feelings and invest your energy in helping care for this patient, whether that is through acting compassionately or through patient-centred management decisions.**

**Session 1 - Screen Nine**

**Exercise Four: Evening Reflection.**

Finally, you are to keep a brief, structured journal of each day, summing up your thoughts and actions. The focus is primarily on what has been unhelpful, what has been left undone that you meant to do that day, and finally a list of things you have done well.

By knowing you will be facing your own judgment in the evening, it was thought that not only would we act better during the day, but we would also be able to act better the next day, and free ourselves from guilt or dwelling on troublesome thoughts and events. This exercise has been chosen to allow us an honest appraisal of our thoughts and actions, and the chance to prepare for troublesome or problematic ways of thinking the next day.

**Session 1 - Screen Ten**

***Instructions:***

Each day you will be asked to complete a reflection from:

- The previous day if you are completing this in the morning.
- The current day if you are completing this in the afternoon/evening.

This exercise is best completed in the evening, so if you are unable to complete this online in the evening, it would be best to complete this exercise on pen and paper and transcribe it into the box the exercise box the following day. If this is not possible then follow the instructions as best you can.

1. Write down a list of thoughts or actions that you were not proud of or were not helpful. For example

- I sent a rude text message to someone when they annoyed me.
- I spent too much time worrying about a piece of coursework and putting it off than working on it.
- I thought unkind things about people I do not know very well.

1. Write down a list of things that you have not done or have left undone that day. For example:

- I did not take the opportunity to stop my unkind thoughts about people I don’t know very well by reminding myself they are human beings just as I am.
- I did not take the opportunity to examine and control my judgements when I was annoyed by someone.

1. Write down a list of things that you have done well that day. For example:

- Today I recognised when I was adding to my judgements in a negative way when a colleague spoke to me, and was able to stop feeling badly about them and not get upset.
- Today I recognised that I desired an expensive gadget for the wrong reasons and resisted the temptation to irresponsibly purchase it.

**Session 1 - Screen Eleven**

**Exercise One: Predicting Misfortune.**

**Instructions**

1. List the activities or actions you will be taking part in that day as well as things you want to achieve.
2. Think of, and write out what misfortune may befall you with regard to each of these items, and how you would cope with it.
3. Think of, and write out what you would do to cope with if each misfortune where to happen.

SPACE FOR TEXT ENTRY

**Session 1 - Screen Twelve**

**Exercise Two: Examine Our Judgements.**

**Instructions**

1. Write down a list of the judgments you have made.
2. Examine each judgement in terms of the sequence of events that led to that interpretation. For example:

SPACE FOR TEXT ENTRY

**Session 1 - Screen Thirteen**

**Exercise Three: Developing empathic reserves**

**Instructions**

- 1. Write a description of a scenario where you may need to offer a patient empathy.
  2. Imagine this scenario proves emotional and you become distressed. Write down how you could feel and why.
  3. This about what could go wrong if you become distressed. List these possibilities.
  4. Imagine the same situation going well, where you do not feel distressed but offered the same empathy. How did you think differently about the scenario?
  5. Examine each thought you had in question 3 and challenge these. Consider what you would do to cope if each of these misfortunes were to happen.

FREE TEXT SPACE

**Exercise Four: Evening Reflection.**

**Instructions**

1. Write down a list of thoughts or actions that you were not proud of or were not helpful.
2. Write down a list of things that you have not done or have left undone that day.
3. Write down a list of things that you have done well that day.

SPACE FOR TEXT ENTRY

**Session 1 - Screen Fourteen**

Thank you for completing today’s session.

Tomorrow will be session 2 of the training.

**Session 2 - Screen One**

Welcome back for the second session of the training.

As before, you will be asked to complete the same exercises as yesterday. Please write as fully with as much detail as you can. Your answers are stored securely and are anonymised.

**Session 2 - Screen Two**

**Exercise One: Predicting Misfortune.**

**Instructions**

1. List the activities or actions you will be taking part in that day as well as things you want to achieve.
2. Think of, and write out what misfortune may befall you with regard to each of these items, and how you would cope with it.
3. Think of, and write out what you would do to cope with if each misfortune where to happen.

SPACE FOR TEXT ENTRY

**Session 2 - Screen Three**

**Exercise Two: Examine Our Judgements.**

**Instructions**

1. Write down a list of the judgments you have made.
2. Examine each judgement in terms of the sequence of events that led to that interpretation. For example:

SPACE FOR TEXT ENTRY

**Session 2 - Screen Four**

**Exercise Three: Developing empathic reserves**

**Instructions**

1. Write a description of a scenario where you may need to offer a patient empathy.
2. Imagine this scenario proves emotional and you become distressed. Write down how you could feel and why.
3. This about what could go wrong if you become distressed. List these possibilities.
4. Imagine the same situation going well, where you do not feel distressed but offered the same empathy. How did you think differently about the scenario?
5. Examine each thought you had in question 3 and challenge these. Consider what you would do to cope if each of these misfortunes were to happen.

FREE TEXT SPACE

**Session 2 - Screen Five**

**Exercise Four: Evening Reflection.**

**Instructions**

1. Write down a list of thoughts or actions that you were not proud of or were not helpful.
2. Write down a list of things that you have not done or have left undone that day.
3. Write down a list of things that you have done well that day.

SPACE FOR TEXT ENTRY

**Session 2 - Screen Six**

Thank you for completing today’s session.

Tomorrow will be session 3 of the training.

**Session 3 - Screen One**

Welcome back for the third session of the training.

As before, you will be asked to complete the same exercises as yesterday. Please write as fully with as much detail as you can. Your answers are stored securely and are anonymised.

**Session 3 - Screen Two**

**Exercise One: Predicting Misfortune.**

**Instructions**

1. List the activities or actions you will be taking part in that day as well as things you want to achieve.
2. Think of, and write out what misfortune may befall you with regard to each of these items, and how you would cope with it.
3. Think of, and write out what you would do to cope with if each misfortune where to happen.

SPACE FOR TEXT ENTRY

**Session 3 - Screen Three**

**Exercise Two: Examine Our Judgements.**

**Instructions**

1. Write down a list of the judgments you have made.
2. Examine each judgement in terms of the sequence of events that led to that interpretation. For example:

SPACE FOR TEXT ENTRY

**Session 3 - Screen Four**

**Exercise Three: Developing empathic reserves**

**Instructions**

1. Write a description of a scenario where you may need to offer a patient empathy.
2. Imagine this scenario proves emotional and you become distressed. Write down how you could feel and why.
3. This about what could go wrong if you become distressed. List these possibilities.
4. Imagine the same situation going well, where you do not feel distressed but offered the same empathy. How did you think differently about the scenario?
5. Examine each thought you had in question 3 and challenge these. Consider what you would do to cope if each of these misfortunes were to happen.

**Session 3 - Screen Five**

**Exercise Four: Evening Reflection.**

**Instructions**

1. Write down a list of thoughts or actions that you were not proud of or were not helpful.
2. Write down a list of things that you have not done or have left undone that day.
3. Write down a list of things that you have done well that day.

SPACE FOR TEXT ENTRY

**Session 3 - Screen Six**

Thank you for completing today’s session.

Tomorrow will be session 4 of the training.

**Session 4 - Screen One**

Welcome back for the fourth session of the training.

As before, you will be asked to complete the same exercises as yesterday. Please write as fully with as much detail as you can. Your answers are stored securely and are anonymised.

**Session 4 - Screen Two**

**Exercise One: Predicting Misfortune.**

**Instructions**

1. List the activities or actions you will be taking part in that day as well as things you want to achieve.
2. Think of, and write out what misfortune may befall you with regard to each of these items, and how you would cope with it.
3. Think of, and write out what you would do to cope with if each misfortune where to happen.

SPACE FOR TEXT ENTRY

**Session 4 - Screen Three**

**Exercise Two: Examine Our Judgements.**

**Instructions**

1. Write down a list of the judgments you have made.
2. Examine each judgement in terms of the sequence of events that led to that interpretation. For example:

SPACE FOR TEXT ENTRY

**Session 4 - Screen Four**

**Exercise Three: Developing empathic reserves**

**Instructions**

1. Write a description of a scenario where you may need to offer a patient empathy.
2. Imagine this scenario proves emotional and you become distressed. Write down how you could feel and why.
3. This about what could go wrong if you become distressed. List these possibilities.
4. Imagine the same situation going well, where you do not feel distressed but offered the same empathy. How did you think differently about the scenario?
5. Examine each thought you had in question 3 and challenge these. Consider what you would do to cope if each of these misfortunes were to happen.

SPACE FOR TEXT ENTRY

**Session 4 - Screen Five**

**Exercise Four: Evening Reflection.**

**Instructions**

1. Write down a list of thoughts or actions that you were not proud of or were not helpful.
2. Write down a list of things that you have not done or have left undone that day.
3. Write down a list of things that you have done well that day.

SPACE FOR TEXT ENTRY

**Session 4 - Screen Six**

Thank you for completing today’s session.

Tomorrow will be session 5 of the training.

**Session 5 - Screen One**

Welcome back for the fifth session of the training.

As before, you will be asked to complete the same exercises as yesterday. Please write as fully with as much detail as you can. Your answers are stored securely and are anonymised.

**Session 5 - Screen Two**

**Exercise One: Predicting Misfortune.**

**Instructions**

1. List the activities or actions you will be taking part in that day as well as things you want to achieve.
2. Think of, and write out what misfortune may befall you with regard to each of these items, and how you would cope with it.
3. Think of, and write out what you would do to cope with if each misfortune where to happen.

SPACE FOR TEXT ENTRY

**Sesson 5 - Screen Three**

**Exercise Two: Examine Our Judgements.**

**Instructions**

1. Write down a list of the judgments you have made.
2. Examine each judgement in terms of the sequence of events that led to that interpretation. For example:

SPACE FOR TEXT ENTRY

**Session 5 - Screen Four**

**Exercise Three: Developing empathic reserves**

**Instructions**

1. Write a description of a scenario where you may need to offer a patient empathy.
2. Imagine this scenario proves emotional and you become distressed. Write down how you could feel and why.
3. This about what could go wrong if you become distressed. List these possibilities.
4. Imagine the same situation going well, where you do not feel distressed but offered the same empathy. How did you think differently about the scenario?
5. Examine each thought you had in question 3 and challenge these. Consider what you would do to cope if each of these misfortunes were to happen.

SPACE FOR TEXT ENTRY

**Session 5 - Screen Five**

**Exercise Four: Evening Reflection.**

**Instructions**

1. Write down a list of thoughts or actions that you were not proud of or were not helpful.
2. Write down a list of things that you have not done or have left undone that day.
3. Write down a list of things that you have done well that day.

SPACE FOR TEXT ENTRY

**Session 5 - Screen Six**

Thank you for completing today’s session.

Tomorrow will be session 6 of the training.

**Session 6 - Screen One**

Welcome back for the sixth session of the training.

As before, you will be asked to complete the same exercises as yesterday. Please write as fully with as much detail as you can. Your answers are stored securely and are anonymised.

**Session 6 - Screen Two**

**Exercise One: Predicting Misfortune.**

**Instructions**

1. List the activities or actions you will be taking part in that day as well as things you want to achieve.
2. Think of, and write out what misfortune may befall you with regard to each of these items, and how you would cope with it.
3. Think of, and write out what you would do to cope with if each misfortune where to happen.

SPACE FOR TEXT ENTRY

**Session 6 - Screen Three**

**Exercise Two: Examine Our Judgements.**

**Instructions**

1. Write down a list of the judgments you have made.
2. Examine each judgement in terms of the sequence of events that led to that interpretation. For example:

SPACE FOR TEXT ENTRY

**Session 6 - Screen Four**

**Exercise Three: Developing empathic reserves**

**Instructions**

1. Write a description of a scenario where you may need to offer a patient empathy.
2. Imagine this scenario proves emotional and you become distressed. Write down how you could feel and why.
3. This about what could go wrong if you become distressed. List these possibilities.
4. Imagine the same situation going well, where you do not feel distressed but offered the same empathy. How did you think differently about the scenario?
5. Examine each thought you had in question 3 and challenge these. Consider what you would do to cope if each of these misfortunes were to happen.

SPACE FOR TEXT ENTRY

**Session 6 - Screen Five**

**Exercise Four: Evening Reflection.**

**Instructions**

1. Write down a list of thoughts or actions that you were not proud of or were not helpful.
2. Write down a list of things that you have not done or have left undone that day.
3. Write down a list of things that you have done well that day.

SPACE FOR TEXT ENTRY

**Session 6 - Screen Six**

Thank you for completing today’s session.

Tomorrow will be session 7 of the training.

**Session 7 - Screen One**

Welcome back for the seventh session of the training.

As before, you will be asked to complete the same exercises as yesterday. Please write as fully with as much detail as you can. Your answers are stored securely and are anonymised.

**Session 7 - Screen Two**

**Exercise One: Predicting Misfortune.**

**Instructions**

1. List the activities or actions you will be taking part in that day as well as things you want to achieve.
2. Think of, and write out what misfortune may befall you with regard to each of these items, and how you would cope with it.
3. Think of, and write out what you would do to cope with if each misfortune where to happen.

SPACE FOR TEXT ENTRY

**Session 7 - Screen Three**

**Exercise Two: Examine Our Judgements.**

**Instructions**

1. Write down a list of the judgments you have made.
2. Examine each judgement in terms of the sequence of events that led to that interpretation. For example:

SPACE FOR TEXT ENTRY

**Session 7 - Screen Four**

**Exercise Three: Developing empathic reserves**

**Instructions**

1. Write a description of a scenario where you may need to offer a patient empathy.
2. Imagine this scenario proves emotional and you become distressed. Write down how you could feel and why.
3. This about what could go wrong if you become distressed. List these possibilities.
4. Imagine the same situation going well, where you do not feel distressed but offered the same empathy. How did you think differently about the scenario?
5. Examine each thought you had in question 3 and challenge these. Consider what you would do to cope if each of these misfortunes were to happen.

SPACE FOR TEXT ENTRY

**Session 7 - Screen Five**

**Exercise Four: Evening Reflection.**

**Instructions**

1. Write down a list of thoughts or actions that you were not proud of or were not helpful.
2. Write down a list of things that you have not done or have left undone that day.
3. Write down a list of things that you have done well that day.

SPACE FOR TEXT ENTRY

**Session 7 - Screen Six**

Thank you for completing today’s session.

Tomorrow will be session 8 of the training.

**Session 8 - Screen One**

Welcome back for the eighth session of the training.

As before, you will be asked to complete the same exercises as yesterday. Please write as fully with as much detail as you can. Your answers are stored securely and are anonymised.

**Session 8 - Screen Two**

**Exercise One: Predicting Misfortune.**

**Instructions**

1. List the activities or actions you will be taking part in that day as well as things you want to achieve.
2. Think of, and write out what misfortune may befall you with regard to each of these items, and how you would cope with it.
3. Think of, and write out what you would do to cope with if each misfortune where to happen.

SPACE FOR TEXT ENTRY

**Session 8 - Screen Three**

**Exercise Two: Examine Our Judgements.**

**Instructions**

1. Write down a list of the judgments you have made.
2. Examine each judgement in terms of the sequence of events that led to that interpretation. For example:

SPACE FOR TEXT ENTRY

**Session 8 - Screen Four**

**Exercise Three: Developing empathic reserves**

**Instructions**

1. Write a description of a scenario where you may need to offer a patient empathy.
2. Imagine this scenario proves emotional and you become distressed. Write down how you could feel and why.
3. This about what could go wrong if you become distressed. List these possibilities.
4. Imagine the same situation going well, where you do not feel distressed but offered the same empathy. How did you think differently about the scenario?
5. Examine each thought you had in question 3 and challenge these. Consider what you would do to cope if each of these misfortunes were to happen.

SPACE FOR TEXT ENTRY

**Session 8 - Screen Five**

**Exercise Four: Evening Reflection.**

**Instructions**

1. Write down a list of thoughts or actions that you were not proud of or were not helpful.
2. Write down a list of things that you have not done or have left undone that day.
3. Write down a list of things that you have done well that day.

SPACE FOR TEXT ENTRY

**Session 8 - Screen Six**

Thank you for completing today’s session.

Tomorrow will be session 9 of the training.

**Session 9 - Screen One**

Welcome back for the seventh session of the training.

As before, you will be asked to complete the same exercises as yesterday. Please write as fully with as much detail as you can. Your answers are stored securely and are anonymised.

**Session 9 - Screen Two**

**Exercise One: Predicting Misfortune.**

**Instructions**

1. List the activities or actions you will be taking part in that day as well as things you want to achieve.
2. Think of, and write out what misfortune may befall you with regard to each of these items, and how you would cope with it.
3. Think of, and write out what you would do to cope with if each misfortune where to happen.

SPACE FOR TEXT ENTRY

**Session 9 - Screen Three**

**Exercise Two: Examine Our Judgements.**

**Instructions**

1. Write down a list of the judgments you have made.
2. Examine each judgement in terms of the sequence of events that led to that interpretation. For example:

SPACE FOR TEXT ENTRY

**Session 9 - Screen Four**

**Exercise Three: Developing empathic reserves**

**Instructions**

1. Write a description of a scenario where you may need to offer a patient empathy.
2. Imagine this scenario proves emotional and you become distressed. Write down how you could feel and why.
3. This about what could go wrong if you become distressed. List these possibilities.
4. Imagine the same situation going well, where you do not feel distressed but offered the same empathy. How did you think differently about the scenario?
5. Examine each thought you had in question 3 and challenge these. Consider what you would do to cope if each of these misfortunes were to happen.

SPACE FOR TEXT ENTRY

**Session 9 - Screen Five**

**Exercise Four: Evening Reflection.**

**Instructions**

1. Write down a list of thoughts or actions that you were not proud of or were not helpful.
2. Write down a list of things that you have not done or have left undone that day.
3. Write down a list of things that you have done well that day.

SPACE FOR TEXT ENTRY

**Session 9 - Screen Six**

Thank you for completing today’s session.

Tomorrow will be session 10 of the training.

**Session 10 - Screen One**

Welcome back for the seventh session of the training.

As before, you will be asked to complete the same exercises as yesterday. Please write as fully with as much detail as you can. Your answers are stored securely and are anonymised.

**Session 10 - Screen Two**

**Exercise One: Predicting Misfortune.**

**Instructions**

1. List the activities or actions you will be taking part in that day as well as things you want to achieve.
2. Think of, and write out what misfortune may befall you with regard to each of these items, and how you would cope with it.
3. Think of, and write out what you would do to cope with if each misfortune where to happen.

SPACE FOR TEXT ENTRY

**Session 10 - Screen Three**

**Exercise Two: Examine Our Judgements.**

**Instructions**

1. Write down a list of the judgments you have made.
2. Examine each judgement in terms of the sequence of events that led to that interpretation. For example:

SPACE FOR TEXT ENTRY

**Session 10 - Screen Four**

**Exercise Three: Developing empathic reserves**

**Instructions**

1. Write a description of a scenario where you may need to offer a patient empathy.
2. Imagine this scenario proves emotional and you become distressed. Write down how you could feel and why.
3. This about what could go wrong if you become distressed. List these possibilities.
4. Imagine the same situation going well, where you do not feel distressed but offered the same empathy. How did you think differently about the scenario?
5. Examine each thought you had in question 3 and challenge these. Consider what you would do to cope if each of these misfortunes were to happen.

SPACE FOR TEXT ENTRY

**Session 10 - Screen Five**

**Exercise Four: Evening Reflection.**

**Instructions**

1. Write down a list of thoughts or actions that you were not proud of or were not helpful.
2. Write down a list of things that you have not done or have left undone that day.
3. Write down a list of things that you have done well that day.

SPACE FOR TEXT ENTRY

**Session 10 - Screen Six**

Thank you for completing today’s session.

Tomorrow will be session 11 of the training.

**Session 11 - Screen One**

Welcome back for the seventh session of the training.

As before, you will be asked to complete the same exercises as yesterday. Please write as fully with as much detail as you can. Your answers are stored securely and are anonymised.

**Session 11 - Screen Two**

**Exercise One: Predicting Misfortune.**

**Instructions**

1. List the activities or actions you will be taking part in that day as well as things you want to achieve.
2. Think of, and write out what misfortune may befall you with regard to each of these items, and how you would cope with it.
3. Think of, and write out what you would do to cope with if each misfortune where to happen.

SPACE FOR TEXT ENTRY

**Session 11 - Screen Three**

**Exercise Two: Examine Our Judgements.**

**Instructions**

1. Write down a list of the judgments you have made.
2. Examine each judgement in terms of the sequence of events that led to that interpretation. For example:

SPACE FOR TEXT ENTRY

**Session 11 - Screen Four**

**Exercise Three: Developing empathic reserves**

**Instructions**

1. Write a description of a scenario where you may need to offer a patient empathy.
2. Imagine this scenario proves emotional and you become distressed. Write down how you could feel and why.
3. This about what could go wrong if you become distressed. List these possibilities.
4. Imagine the same situation going well, where you do not feel distressed but offered the same empathy. How did you think differently about the scenario?
5. Examine each thought you had in question 3 and challenge these. Consider what you would do to cope if each of these misfortunes were to happen.

SPACE FOR TEXT ENTRY

**Session 11 - Screen Five**

**Exercise Four: Evening Reflection.**

**Instructions**

1. Write down a list of thoughts or actions that you were not proud of or were not helpful.
2. Write down a list of things that you have not done or have left undone that day.
3. Write down a list of things that you have done well that day.

SPACE FOR TEXT ENTRY

**Session 11 - Screen Six**

Thank you for completing today’s session.

Tomorrow will be session 12 of the training.

**Session 12 - Screen One**

Welcome back for the seventh session of the training.

As before, you will be asked to complete the same exercises as yesterday. Please write as fully with as much detail as you can. Your answers are stored securely and are anonymised.

**Session 12 - Screen Two**

**Exercise One: Predicting Misfortune.**

**Instructions**

1. List the activities or actions you will be taking part in that day as well as things you want to achieve.
2. Think of, and write out what misfortune may befall you with regard to each of these items, and how you would cope with it.
3. Think of, and write out what you would do to cope with if each misfortune where to happen.

SPACE FOR TEXT ENTRY

**Session 12 - Screen Three**

**Exercise Two: Examine Our Judgements.**

**Instructions**

1. Write down a list of the judgments you have made.
2. Examine each judgement in terms of the sequence of events that led to that interpretation. For example:

SPACE FOR TEXT ENTRY

**Session 12 - Screen Four**

**Exercise Three: Developing empathic reserves**

**Instructions**

1. Write a description of a scenario where you may need to offer a patient empathy.
2. Imagine this scenario proves emotional and you become distressed. Write down how you could feel and why.
3. This about what could go wrong if you become distressed. List these possibilities.
4. Imagine the same situation going well, where you do not feel distressed but offered the same empathy. How did you think differently about the scenario?
5. Examine each thought you had in question 3 and challenge these. Consider what you would do to cope if each of these misfortunes were to happen.

SPACE FOR TEXT ENTRY

**Session 12 - Screen Five**

**Exercise Four: Evening Reflection.**

**Instructions**

1. Write down a list of thoughts or actions that you were not proud of or were not helpful.
2. Write down a list of things that you have not done or have left undone that day.
3. Write down a list of things that you have done well that day.

SPACE FOR TEXT ENTRY

**Session 12 - Screen Six**

Thank you for completing today’s session, and the required number of training sessions!
