# Supplementary material for: Can stoic training develop medical student empathy and resilience? A mixed-methods study
Source: BMC Med Educ. 2022 May 3;22:340. doi: 10.1186/s12909-022-03391-x (PMC9064267; doi:10.1186/s12909-022-03391-x)
Supplement: Supplementary file 2 — Additional file 2. [file 12909_2022_3391_MOESM2_ESM.docx]

**Additional file 2**

**Semi-structured interview question stems**

Interview 1:

| **Demographic information** | - Age  - Gender  - Ethnicity  -Sexual orientation  (Inform can decline to answer/ state they prefer not to say). |
| --- | --- |
| **Understanding experiences in using the training package** | -Can you tell me about how you found using the training package?  -What did you like about the package?  -What worked about the package?  -What didn’t you like about the package?  -What didn’t work about the package?  -Was there anything you didn’t understand or found confusing?  -Do you have any take home messages from the training? If so, what are these? If not, do you think there are any reasons why it hasn’t left a lasting impression?  -Do you think this training will influence your practice as a medical student? If yes, how + why? If no- why not? |
| **Understanding current empathy** | -Can you tell me more about how you feel about practicing empathy as a medical student?  -Can you tell me about a time recently where you offered empathy?  -Can you tell me about a time recently when empathy was difficult to give?  -Do you think this training will influence how you practice empathy? If so, how and why? If no, why not?  -Were there any ways the training could have been improved specifically in regard to empathy? |
| **Understanding current resilience** | -What do you think about the concept of resilience?  -How do you try to be resilient/maintain resilience?  -Can you tell me about a time recently where you had to be resilient?  -Do you think this training will influence how you practice resilience? If so, how and why? If no, why not?  -Were there any ways the training could have been improved specifically in regard to resilience? |
| **Concluding the interview** | -Do you have any further comments or examples about use of the training package that you feel are important and we haven’t covered?  -Is there anything you would expand upon that we haven’t spoken about? |
| **Thanks, and debriefing** |  |

Interview 2:

| **Understanding experiences in using the training package** | -Do you have any lasting impression from the training you did 2 months ago?  -If yes, what is your lasting impression/what are your lasting impressions? Why do you think these have stayed with you?  -If no, why do you think there hasn’t been a lasting impact? Could anything have been changed about the training to make it more memorable?  -Have you thought about the training since using it?  -Have you used any aspects of this training in your practice as a medical student since? Examples + how this went if so. |
| --- | --- |
| **Understanding current resilience** | -Since we last spoke, can you tell me about a time where you had to be resilient?  -Any influence of training package?  -Desire for future focus |
| **Understanding current empathy** | -Since we last spoke, can you tell me about a time where you felt positively about the empathy you gave?  -Since we last spoke, can you tell me about a time when you felt negatively about the empathy you gave?  -How have you felt in handling emptions borne of empathy since we last spoke?  -Any influence of training package?  -Desire for future focus |
| **Concluding the interview** | -Do you have any further comments or examples about use of the training package that you feel are important and we haven’t covered?  -Is there anything you would expand upon that we haven’t spoken about? |
| **Thanks, and debriefing** |  |
